# Cerium Salts: An Efficient Curing Catalyst for Benzoxazine Based Coatings

**DOI:** 10.3390/polym12020415

**Published:** 2020-02-11

**Authors:** Tao Zhang, Leïla Bonnaud, Jean-Marie Raquez, Marc Poorteman, Marjorie Olivier, Philippe Dubois

**Affiliations:** 1 Laboratory of Polymeric and Composite Materials, Center of Innovation and Research in Materials and Polymers (CIRMAP), Materia Nova Research Center & University of Mons, 23 Place du Parc, B-7000 Mons, Belgium; taoyuan0510@126.com (T.Z.); jean-marie.raquez@umons.ac.be (J.-M.R.); philippe.dubois@umons.ac.be (P.D.); 2 Department of Visual Communication Design, School of Art & Design, Zhejiang Sci-Tech University, Hangzhou 310018, China; 3 Department of Materials Science, Materials Engineering Research Center (CRIM), University of Mons, 23 Place du Parc, B-7000 Mons, Belgium; Marc.POORTEMAN@umons.ac.be (M.P.); marjorie.olivier@umons.ac.be (M.O.)

**Keywords:** benzoxazine, cerium salt, ROP, catalyst, low curing temperature

## Abstract

The effect of three different cerium salts (Ce(NO_3_)_3_·6H_2_O, CeCl_3_·7H_2_O and Ce(OOCCH_3_)_3_·5H_2_O) on the ring-opening polymerization (ROP) of a model diamine-based benzoxazine (4EP-pPDA) was investigated. With the incorporation of the cerium salts, the curing temperature of 4EP-pPDA is reduced substantially, and the glass transition temperatures of the resulting networks are increased significantly. The three cerium salts exhibit different catalytic activities, which were analyzed by FT-IR, NMR, and energy-dispersive X-ray (EDX). Ce(NO_3_)_3_·6H_2_O was found to exhibit the best catalytic effect, which seems to be related to its better dispersibility within 4EP-pPDA benzoxazine precursors.

## 1. Introduction

Benzoxazine resins are relatively new incomers in the field of thermosetting materials [1,2] and they are obtained from the ring-opening polymerization of 1,3-benzoxazine precursors [3]. The latter can be straightforwardly synthesized in high yield (>90wt%) by a Mannich-like condensation of an amine, a phenol, and formaldehyde, either in solution or in bulk [4,5,6]. Moreover, the diverse and large number of commercially available phenolic derivatives and amine derivatives allows the preparation of a very wide range of monomers including the incorporation of additional substituents which can bring new functionalities to materials such as self-healing [7], self-cleaning [8], photo-sensing [9] properties, etc. Benzoxazine resins have attracted substantial attention these last two decades in the first place, it is mainly because they exhibit a combination of excellent properties [10,11] such as a high glass transition temperature, near-zero volumetric change upon curing, low water absorption, good electrical resistance, low surface free energy, and high char yield. Many endeavors have been made to explore the possible applications in the automotive, aerospace, and construction industries in the form of matrix resin or surface coating [8,12,13]. However, for most benzoxazine precursors, their thermal ring-opening polymerization usually occurs at a high curing temperature (i.e., over 180 °C) not compatible with several manufacturing processes of materials. This drawback could limit the use of benzoxazine, and it particularly cannot withstand such high temperature heating as surface coatings for several substrates [14,15,16]. Therefore, the search for reaction paths reducing the curing temperature has become one of the main focuses to overcome in this field.

Although it is not fully elucidated, it is commonly accepted that the thermal ring-opening polymerization of benzoxazine monomers occurs via cationic polymerization and the mechanism includes mainly three steps: coordination ring-opening of the oxazine ring, an electrophilic attack, and, finally, rearrangement [17]. In this context, several strategies have been proposed and reported in the literature to lower the polymerization temperature of benzoxazine precursors.

First, thanks to the great flexibility in the design of the molecular structure of benzoxazine monomers, several studies have focused on the understanding of the effects of structural parameters—including the nature, position, and hindrance that chemical groups bear on the aromatic backbone—on the reactivity of the corresponding monomers. For instance, the comparison of structurally different phenylenediamine-based benzoxazines with resorcinol/hydroquinone-based benzoxazines has indicated that the curing exotherm seems to be influenced to a large extent by the position of the oxazine ring on the aromatic units [18]. The influence of bridging groups on thermal ring-opening polymerization was also studied on diphenol-based benzoxazine monomers [19]. The authors observed that electron-withdrawing groups promoted thermal activated polymerization and that the curing temperature decreased by lowering the bond energy of C–O on oxazine rings. The distance separating the oxazine groups also seems an important parameter in the case of linear aliphatic diamine-based benzoxazine monomers. Indeed, it appears that the polymerization exotherm position decreases to a lower temperature range with the shortening of the length of the aliphatic diamine chain [20]. Moreover, the synthesis of multifunctional benzoxazines is also reported to undergo polymerization at lower temperatures when the number of oxazine functionalities increases [21,22].

In addition to these structural considerations, it also appears that the introduction of certain appropriate moieties like hydroxyl [23,24], imidazole [25], and amine [26] groups onto the backbone structure of benzoxazine monomers lowers the temperature of the ring-opening of the oxazine. In fact, it is now well known that the introduction of electron-withdrawing or electron-donating groups on the structural skeleton of benzoxazine monomers can greatly affect their reactivity by activating the ring-opening or stabilizing the intermediates [27]. Indeed, the catalyst effect of neighboring groups accelerating benzoxazine polymerization has been clearly shown in the case of carboxylic acid- [28], hydroxyl- [29], and amide [30] containing benzoxazine monomers. Moreover, the substitution on the ortho-position was found to further stimulate and activate the ring-opening polymerization when compared to its para-substituted counterpart as well in the case of a methylol functional benzoxazine [23,31] as in the case of an ortho-amide-imide functional benzoxazine [30].

Another strategy uses the reactivity of benzoxazine functions towards other functional groups such as amines. This approach is a rather new polymerization method via copolymerization than a direct acceleration of benzoxazine polymerization. Nevertheless, it is worth noting that in the case of copolymerization of benzoxazines with amines, in addition to the significant lowering of the polymerization temperature, an enhancement of the resulting mechanical and thermal properties of the networks has been achieved [32]. Another study reported the preparation and use of a polyamine-based oligomer to accelerate benzoxazine polymerization. Again, in this case, the polyamine was able to promote both the curing and, simultaneously, enhance the glass transition temperature as well as the thermal stability of the cured network [33].

A third strategy to reduce the curing temperature of benzoxazines consists of using initiators of cationic ring-opening polymerization. Some researchers have employed Lewis acids and nucleophilic catalysts to promote polymerization of benzoxazine, consequently reducing the curing temperature [34,35,36]. Indeed, several Lewis acids such as PCl_5_, PCl_3_, and POCl_3_, and transition metal salts such as TiCl_4_, AlCl_3_, CuCl_2_, AgCl, ZnCl_2_, NiCl_2_, FeCl_3_, and LiI have been tested successfully. Moreover, Liu et al. observed that LiI was especially efficient [37]. Recently, Akkus et al. highlighted that for amine salts, the nucleophilicity of the counterion had high impact, and they observed that the anion I^−^ exhibited the best catalytic behavior [38]. Organic-based catalysts such as acetylacetonato complexes with iron, manganese, or cobalt, sulfonates such as para-toluene sulfonate, or organic oniums such as sulfonium hexafluoroantimonates were also found to be highly efficient for promoting the polymerization of benzoxazine precursors at a lower temperature [39,40,41].

Although many approaches have already been considered, they do not always allow retaining or improving the properties of the pristine polybenzoxazine network. Therefore, there is still a need for developing alternative solutions, which, in addition, should also consider environmental and toxicological issues for practical use, especially in the coating industry.

In this paper, we report the synthesis of a new diamine-based benzoxazine (4EP-pPDA) synthesized by the reaction of bio-basable reactants (4-ethylphenol, 1,4-phenylenediamine, and paraformaldehyde) and its thermal ring-opening polymerization in the presence of different kinds of cerium salts. The structure chosen for the benzoxazine monomers is based on both environmental and theoretical criteria. Indeed, 4-ethylphenol is a biomass-derived phenolic compound which can be obtained following an enzymatic process from natural and widely spread *p*-coumaric acid [42]. In addition to its bio-based character, the interest of 4-ethylphenol structures also lies in the absence of additional reactive functions on its structural backbone, thus allowing to use it as a model molecule to focus exclusively on the effect of cerium salts on the polymerization of benzoxazine functions. To the best of our knowledge, the ability of these metallic salts to accelerate benzoxazine polymerization has never been tested.

## 2. Materials and Methods 

### 2.1. Materials

4-ethylphenol (≥97.0%) and CeCl_3_·7H_2_O (≥99.0%) were purchased from Alfa Aesar (Karlsruhe, Germany). 1,4-phenylenediamine (≥99.0%) and Ce(OOCCH_3_)_3_·5H_2_O (≥99.9%) were purchased from Sigma-Aldrich (Darmstadt, Germany). Ce(NO_3_)_3_·6H_2_O was purchased from Honeywell Fluka (Seelze, Germany). Paraformaldehyde (≥95.0%) and absolute ethanol (≥99.5%) were purchased from VWR (Leuven, Belgium). The starting reagents were used without further purification.

### 2.2. Preparation of 4EP-pPDA

The synthesis of 4EP-pPDA was adapted from a procedure in bulk reported elsewhere by Ishida et al. [4]. 4-ethylphenol 23.21 g (1.9 × 10^−1^ mol) and 1,4-phenylenediamine 10.00 g (9.25 × 10^−2^ mol) were mixed in a beaker with a mechanical stirrer at 120 °C until a homogeneous liquid was obtained. Then, paraformaldehyde in excess of 12.22 g (4.07 × 10^−1^ mol) was rapidly added under vigorous stirring to prevent bubbling from the rapid decomposition of paraformaldehyde into formaldehyde. The resulting mixture was reacted for seven additional minutes under continuous stirring. The crude reaction product was dissolved in refluxing ethanol (about 500 mL) and the precursors of the resin were precipitated upon cooling. The resulting precipitate was collected, filtered and abundantly washed with cold ethanol. Then it was dried under vacuum. A vitrified yellow resin was obtained (weight yield about 65%).

### 2.3. Preparation of 4EP-pPDA/Cerium Salt Mixtures

4EP-pPDA and cerium salts were first added into the dichloromethane and ethanol mixture solvents (dichloromethane/ethanol=90/10 v/v). After stirring for 2 h at room temperature, the solvent was removed by volatilizing and drying under vacuum. The content of each cerium salt in the 4EP-pPDA substrate was 5 mmol/100 g. In this work, we denote briefly the samples made from Ce(NO_3_)_3_·6H_2_O, CeCl_3_·7H_2_O, and Ce(OOCCH_3_)_3_·5H_2_O as 5mM Ce(NO_3_)_3_·6H_2_O, 5mM CeCl_3_·7H_2_O, and 5mM Ce(OOCCH_3_)_3_·5H_2_O, respectively.

### 2.4. Measurements and Characterization

Fourier transform infrared (FT-IR) spectra were recorded using a BRUKER IFS 66v/S spectrometer (Bruker Instruments, Billerica, MA, USA) from 400 to 4000 cm^−1^ with a resolution of 4 cm^−1^. ^1^H-NMR spectra were obtained by using a BRUKER Avance DMX-500 MHZ spectrometer (Bruker Instruments, Billerica, MA, USA) in CDCl_3_ with TMS as the internal standard. Differential scanning calorimetry (DSC) was performed on a TA DSC Q200 calorimeter (TA Instruments, New Castle, DE, USA) from room temperature up to 300 °C with a heating rate of 10 °C/min and a nitrogen flow rate of 50 mL/min. Field-emission scanning electron microscopy (FE-SEM) and energy-dispersive X-ray (EDX) analysis were performed with a Hitachi S-4800 analyzer (Hitachi, Tokyo, Japan) operated at 20 kV. Thermogravimetric analysis (TGA) was performed on a TA TGA Q50 thermogravimetric analyzer (TA Instruments, New Castle, DE, USA) from room temperature to 550 °C with a heating rate of 10 °C/min under a nitrogen atmosphere UV-visible spectral measurements were performed on the elaborated powders with a Labsphere RSA-PE-19 reflectance accessory (Labsphere, N. Sutton, NY, USA).

## 3. Results and Discussion

**Scheme 1:**
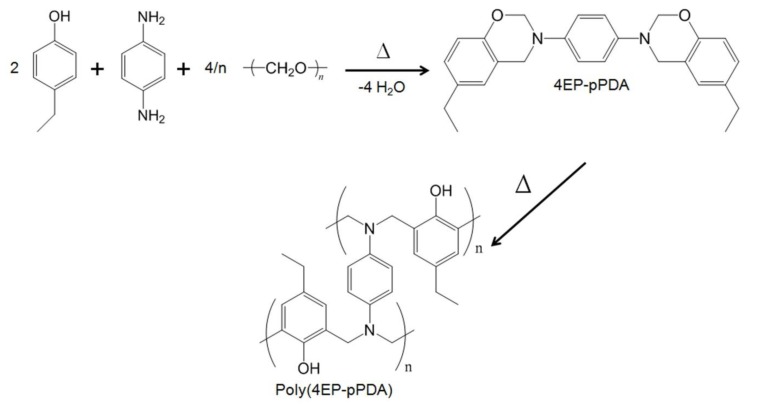
Synthesis of 4EP-pPDA and corresponding cured network of Poly(4EP-pPDA).

A new diamine-based benzoxazine (4EP-pPDA) was synthesized by the reaction of 4-ethylphenol, 1,4-phenylenediamine, and paraformaldehyde as shown in Scheme 1. 

**Figure 1:**
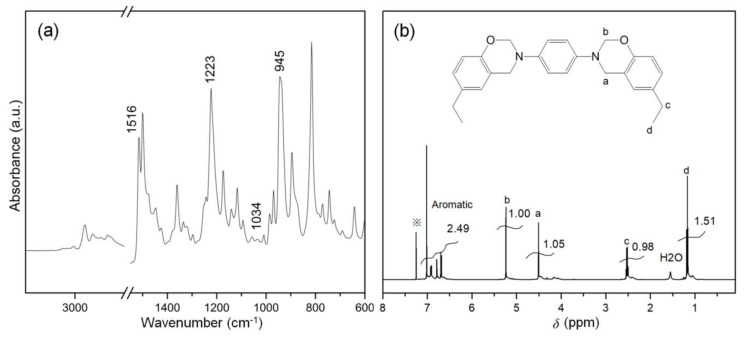
(**a**) FT-IR and (**b**) ^1^H-NMR spectrum of 4EP-pPDA.

The chemical structure was investigated by FT-IR and 1H-NMR. The results are gathered in Figure 1. More specifically, the FT-IR spectrum of 4EP-pPDA shows absorption peaks at 1516, 1223, 1034, and 945 cm^−1^. Based on similar benzoxazine derivatives reported in the literature [43,44,45], they are ascribed to the trisubstituted benzene ring stretching, asymmetric stretching vibration of C-O-C, symmetric stretching vibration of C-O-C, and out-of-plane C-H, respectively. In the ^1^H-NMR spectrum of 4EP-pPDA, the characteristic protons appear at around 4.51 (Ar-CH_2_-N) and 5.24 (O-CH_2_-N) ppm, confirming the formation of the 4EP-pPDA molecular structure [45,46,47]. Furthermore, the integration ratios of the peaks agree well with the proposed chemical structure. These results further confirm the successful synthesis of 4EP-pPDA precursors.

**Scheme 2:**
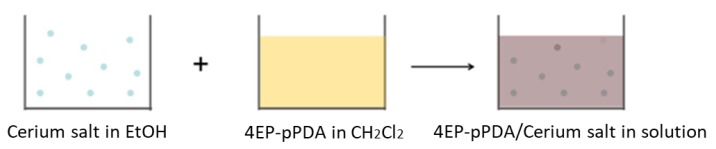
Illustration of the preparation process of 4EP-pPDA/cerium salt mixtures.

Thereafter, the cerium salts were incorporated within the 4EP-pPDA precursors via a solvent mixture of dichloromethane/ethanol (90/10 v/v) as shown in Scheme 2. 

**Figure 2:**
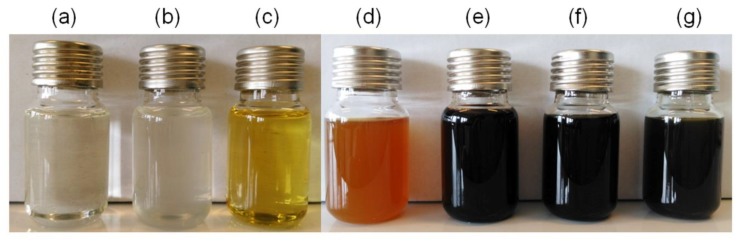
Photos of (**a**) Ce(NO_3_)_3_·6H_2_O, (**b**) CeCl_3_·7H_2_O, (**c**) Ce(OOCCH_3_)_3_·5H_2_O, (**d**) 4EP-pPDA, (**e**) 5 mM Ce(NO_3_)_3_·6H_2_O, (**f**) 5 mM CeCl_3_·7H_2_O, and (**g**) 5mM Ce(OOCCH_3_)_3_·5H_2_O in the solvents of dichloromethane/ethanol (90/10 v/v).

Interestingly, the three salts (i.e., Ce(NO_3_)_3_·6H_2_O, CeCl_3_·7H_2_O, and Ce(OOCCH_3_)_3_·5H_2_O) seem to dissolve well within the 4EP-pPDA-based solution and color changes are observed for the three systems. Indeed, the solutions containing 5 mM Ce(NO_3_)_3_·6H_2_O, 5 mM CeCl_3_·7H_2_O, and 5 mM Ce(OOCCH_3_)_3_·5H_2_O all become dark red (see Figure 2). This color change phenomenon is mostly due to the complexation between a 4EP-pPDA monomer with Ce ions via an oxygen or nitrogen atom in the oxazine ring as coordination atoms [48,49].

**Figure 3:**
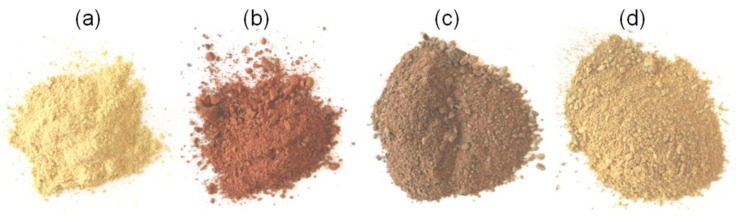
Photos of powders of (**a**) 4EP-pPDA, (**b**) 5 mM Ce(NO_3_)_3_·6H_2_O, (**c**) 5 mM CeCl_3_·7H_2_O, and (**d**) 5 mM Ce(OOCCH_3_)_3_·5H_2_O.

After removal of the solvent mixture, the color of the samples is modified and different shades between the three samples appear, as shown in Figure 3. Interestingly, the color of all samples looks uniform and homogeneous, suggesting a fine dispersion of all the cerium salts within the 4EP-pPDA precursors.

**Figure 4:**
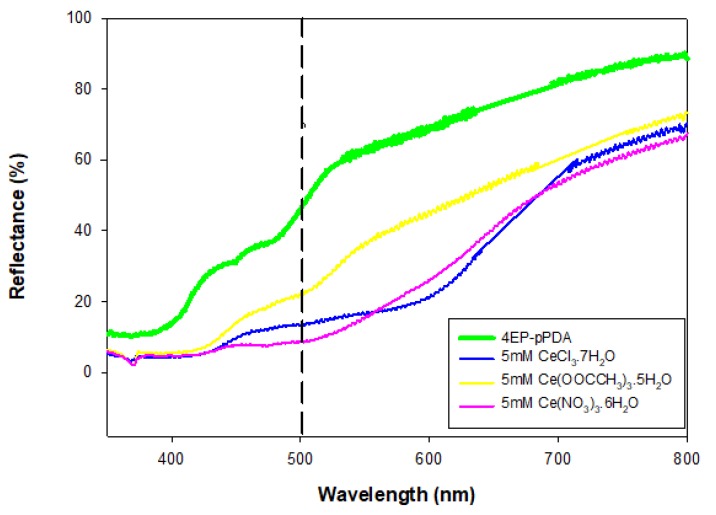
UV-vis spectra of 4EP-pPDA, 5 mM Ce(NO_3_)_3_·6H_2_O, 5 mM CeCl_3_·7H_2_O, and 5 mM Ce(OOCCH_3_)_3_·5H_2_O.

The complexation between 4EP-pPDA monomers with Ce ions was further confirmed by UV-VIS spectroscopy. Indeed, as shown in Figure 4, a broadening and shifting of the band towards 500 nm attributed to the formation of cerium complexes with 4EP-pPDA monomers are observed in the case of systems 5 mM Ce(NO_3_)_3_·6H_2_O, 5 mM CeCl_3_·7H_2_O, and 5 mM Ce(OOCCH_3_)_3_·5H_2_O.

**Figure 5:**
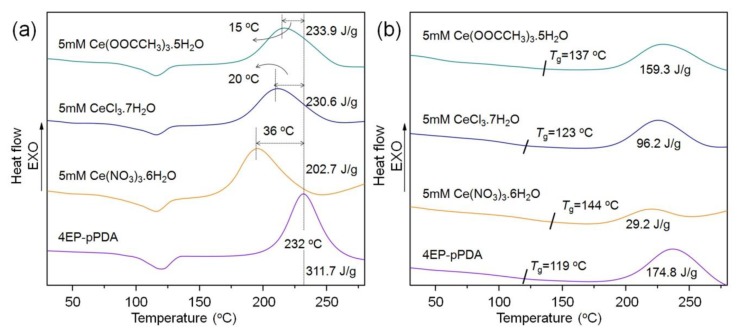
Differential scanning calorimetry (DSC) curves of 5 mM Ce(OOCCH_3_)_3_·5H_2_O, 5 mM CeCl_3_·7H_2_O, 5 mM Ce(NO_3_)_3_·6H_2_O, and 4EP-pPDA (**a**) before and(**b**) after curing at 150 °C for 4 h.

**Figure 6:**
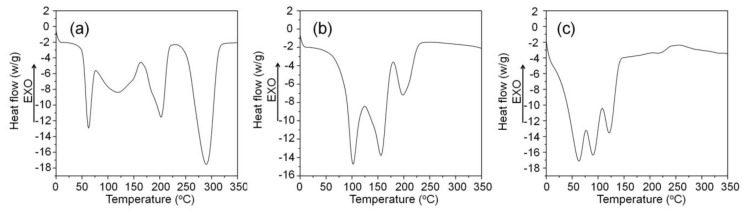
DSC curves of (**a**) Ce(NO_3_)_3_·6H_2_O, (**b**) CeCl_3_·7H_2_O, and (**c**) Ce(OOCCH_3_)_3_·5H_2_O.

The effect of cerium salts on the curing behavior of 4EP-pPDA precursors was studied by DSC, as shown in Figure 5a. The neat 4EP-pPDA precursors synthesized for this study exhibit an exothermic peak with a maximum temperature (*T*_p_) at about 232 °C, corresponding to the ring-opening polymerization of benzoxazine. For all 4EP-pPDA systems containing cerium salt, one exotherm is also observed, but the *T*_p_ of these systems is shifted to lower temperatures. The presence of only one exotherm further confirms the good dispersion of cerium salts within the 4EP-pPDA matrix. It also shows the ability of cerium salts to accelerate the ring-opening polymerization of 4EP-pPDA benzoxazine precursors. Although all three systems contain the same amount of cerium, the decrease in *T*_p_ appears not to be identical. Indeed, the following order is observed: 5 mM Ce(NO_3_)_3_·6H_2_O (36 °C) > 5 mM CeCl_3_·7H_2_O (20 °C) > 5mM Ce(OOCCH_3_)_3_·5H_2_O (15 °C), indicating differences in the catalytic activities for the three cerium salts. On the other hand, an obvious decrease in the enthalpy of the curing reaction (Δ*H*) is observed with the introduction of cerium salt. It also should be noted that Ce(NO_3_)_3_·6H_2_O, CeCl_3_·7H_2_O, and Ce(OOCCH_3_)_3_·5H_2_O all contain structural water which is released in the curing region of 4EP-pPDA, as observed by DSC in Figure 6. Hence, the reason for the reduction of Δ*H* can also be explained by the superposition of two antagonist phenomena, i.e., the benzoxazine polymerization, which is exothermic, and the releasing of crystal water, which is endothermic.

To try and clarify the activities of cerium salts, the 4EP-pPDA systems containing cerium salts were further monitored by DSC after isothermal curing at 150 °C for 4h, as shown in Figure 5b. The polymerization enthalpy Δ*H* of a neat 4EP-pPDA system is found to decrease by 44%. By contrast, the enthalpies of 5 mM Ce(NO_3_)_3_·6H_2_O, 5 mM CeCl_3_·7H_2_O, and 5 mM Ce(OOCCH_3_)_3_·5H_2_O are found to decrease by 86%, 58%, and 32%, respectively. This result clearly indicates that among the three cerium salts, Ce(NO_3_)_3_·6H_2_O is the one exhibiting the best catalytic efficiency, which can significantly shorten the curing time. Surprisingly, Ce(OOCCH_3_)_3_·5H_2_O exhibits the worst performance whereas the activity of CeCl_3_·7H_2_O stands in between that of Ce(NO_3_)_3_·6H_2_O and Ce(OOCCH_3_)_3_·5H_2_O. Furthermore, the order of glass transition temperatures (*T*_g_) observed is 5 mM Ce(NO_3_)_3_·6H_2_O (144 °C) > 5 mM Ce(OOCCH_3_)_3_·5H_2_O (137 °C) > 5 mM CeCl_3_·7H_2_O (123 °C) > 4EP-pPDA (119 °C). The highest *T*_g_ of the 5 mM Ce(NO_3_)_3_·6H_2_O system can be explained by the highest curing degree and the formation of the corresponding polybenzoxazine network.

**Figure 7:**
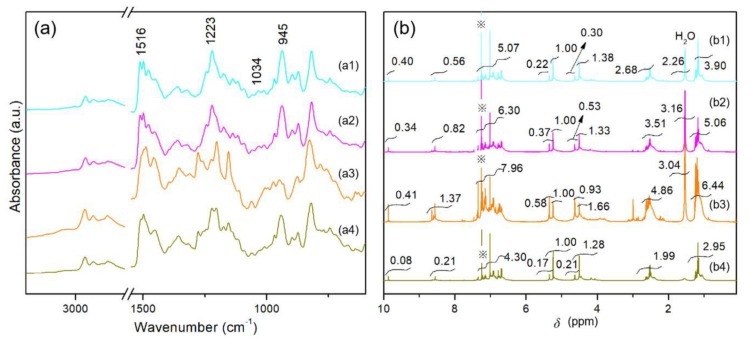
(**a**) FT-IR spectra and (**b**) ^1^H-NMR spectra of the 5mM Ce(OOCCH_3_)_3_·5H_2_O (a1, b1), 5mM CeCl_3_·7H_2_O (a2, b2), 5mM Ce(NO_3_)_3_·6H_2_O (a3, b3), and neat 4EP-pPDA (a4, b4) after curing at 150 °C for 1 h.

To better understand the above results, representative samples of 4EP-pPDA and 4EP-pPDA modified by cerium salts after curing at 150 °C for 1h were analyzed by FT-IR and ^1^H-NMR. As shown in Figure 7a, by selecting the peak at 1516 cm^−1^ as the reference, the relative absorption intensities of the benzoxazine ring for 4EP-pPDA/cerium salt mixtures are found to be weaker than those of 4EP-pPDA. In addition, as presented in Figure 7b, the peaks at 5.24 and 4.51 ppm are split into two signals, and the integrations at 5.24 ppm are lower than those of the samples before curing, indicating the breakage of O-CH_2_-N groups. The order of relative content for the O-CH_2_-N group is 5 mM Ce(NO_3_)_3_·6H_2_O < 5 mM Ce(OOCCH_3_)_3_·5H_2_O < 5 mM CeCl_3_·7H_2_O < 4EP-pPDA. Furthermore, new signals at about 9.9 and 8.5 ppm can be observed, revealing the presence of an Ar-OH group [48]. The order of relative content for the Ar-OH group is 5 mM Ce(NO_3_)_3_·6H_2_O > 5 mM Ce(OOCCH_3_)_3_·5H_2_O > 5 mM CeCl_3_·7H_2_O > 4EP-pPDA. Therefore, from these results it appears clearly that during the curing process the cerium salts promote the breaking of the O-CH_2_-N group and the corresponding cross-linking reaction.

**Figure 8:**
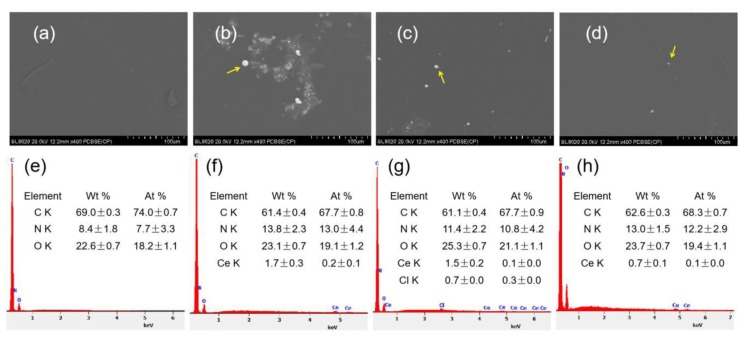
View of SEM images (first row) and EDX spectra (second row) of (**a,e**) 4EP-pPDA, (**b,f**) 5 mM Ce(NO_3_)_3_·6H_2_O, (**c,g**) 5 mM CeCl_3_·7H_2_O, and (**d,h**) 5 mM Ce(OOCCH_3_)_3_·5H_2_O after curing at 150 °C for 4 h.

Figure 8a–d presents the top view SEM images of 4EP-pPDA (a), 5 mM Ce(NO_3_)_3_·6H_2_O (b), 5 mM CeCl_3_·7H_2_O (c), and 5 mM Ce(OOCCH_3_)_3_·5H_2_O (d) after curing at 150 °C for 4 h. Clearly, some white particles can be seen in Figure 8c,d, which are attributed to the cerium salts. The elemental composition of the cured samples was also analyzed as shown in Figure 8e–h. The order of weight content of Ce is 5 mM Ce(NO_3_)_3_·6H_2_O > 5 mM CeCl_3_·7H_2_O > 5 mM Ce(OOCCH_3_)_3_·5H_2_O, which is consistent with the catalytic activity previously determined. The lowest cerium content observed for the sample 5 mM Ce(OOCCH_3_)_3_·5H_2_O is mostly due to the lowest dispersibility of Ce(OOCCH_3_)_3_·5H_2_O. When removing the solvent, most of the Ce(OOCCH_3_)_3_·5H_2_O possibly precipitates out of 4EP-pPDA matrix. Hence, the differences in the catalytic activity are mostly due to the dispersibility of the cerium salt within the 4EP-pPDA matrix. If it is dispersed in the matrix at a molecular level, the complexation between the 4EP-pPDA monomer with Ce ion via the oxygen or nitrogen atom in the oxazine ring may more easily promote the breaking of a O-CH_2_-N group, so the catalytic effect is obvious.

**Figure 9:**
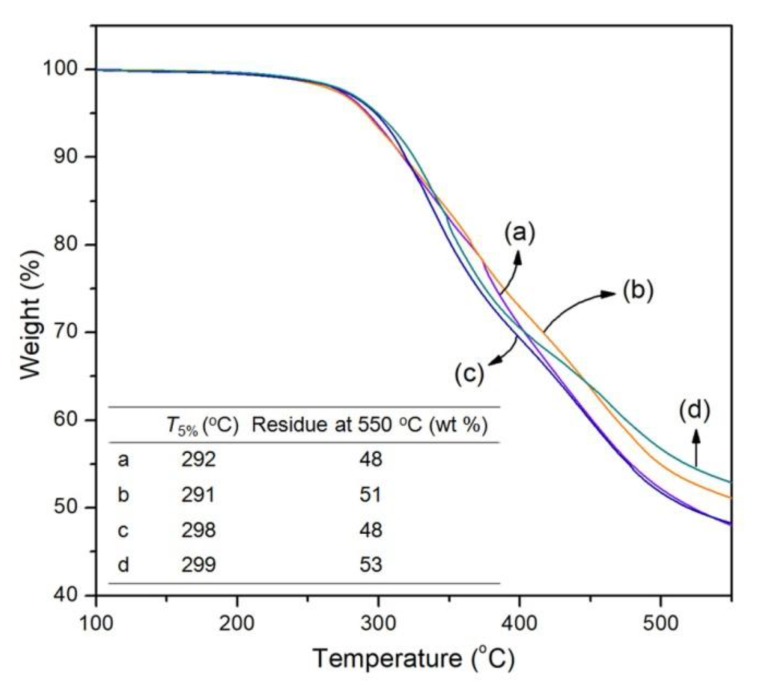
TGA curves of (**a**) 4EP-pPDA, (**b**) 5 mM Ce(NO_3_)_3_·6H_2_O, (**c**) 5 mM CeCl_3_·7H_2_O, and (**d**) 5 mM Ce(OOCCH_3_)_3_·5H_2_O after curing at 150 °C for 4 h.

In addition, the effect of the above three cerium salts on the thermal stability of 4EP-pPDA after curing at 150 °C for 4 h was investigated, as shown in Figure 9. As for the 5 wt % weight loss temperature (*T*_5%_), the Ce(NO_3_)_3_·6H_2_O shows almost no influence, but the CeCl_3_·7H_2_O and Ce(OOCCH_3_)_3_·5H_2_O present obvious improvement. As for the residue at 550 °C, the CeCl_3_·7H_2_O shows no influence, and the Ce(NO_3_)_3_·6H_2_O and Ce(OOCCH_3_)_3_·5H_2_O present little improvement. Therefore, the three cerium salts reported herein can accelerate the ring-opening polymerization of 4EP-pPDA benzoxazine precursors without sacrificing their thermal stability. 

## 4. Conclusions

Model benzoxazine precursors (4EP-pPDA) were synthesized from bio-basable 4-ethylphenol, 1,4-phenylenediamine, and paraformaldehyde and used to test the ability of three different cerium salts (i.e., Ce(OOCCH_3_)_3_·5H_2_O, CeCl_3_·7H_2_O, and Ce(NO_3_)_3_·6H_2_O) to catalyze its ring-opening polymerization. Although the three cerium salts studied were found to accelerate the opening of a O-CH_2_-N group and promote the corresponding cross-linking reaction of 4EP-pPDA precursors, they also exhibit different catalytic activities. Ce(NO_3_)_3_·6H_2_O was found to be the most efficient catalyst to reduce the curing temperature of 4EP-pPDA precursors. This result was mainly related to its better dispersibility within the benzoxazine matrix. It seems that the most important parameter is to obtain a dispersion of the initiator at a molecular scale to achieve optimal catalytic efficiency.
